# Sequencing bias: comparison of different protocols of MicroRNA library construction

**DOI:** 10.1186/1472-6750-10-64

**Published:** 2010-09-06

**Authors:** Geng Tian, XuYang Yin, Hong Luo, XiaoHong Xu, Lars Bolund, XiuQing Zhang

**Affiliations:** 1 Beijing Institute of Genomics, Chinese Academy of Science, Beijing 101300, China; 2 The Graduate University of Chinese Academy of Sciences, Beijing 100062, China; 3 Genome Research Institute, ShenZhen University Medical School, ShenZhen 518000, China; 4 Beijing Genomics Institute, Shenzhen 518000, China; 5 Insitute of Human Genetics, University of Aarhus, Aarhus DK-8000, Denmark

## Abstract

**Background:**

MicroRNAs(miRNAs) are 18-25 nt small RNAs playing critical roles in many biological processes. The majority of known miRNAs were discovered by conventional cloning and a Sanger sequencing approach. The next-generation sequencing (NGS) technologies enable in-depth characterization of the global repertoire of miRNAs, and different protocols for miRNA library construction have been developed. However, the possible bias between the relative expression levels and sequences introduced by different protocols of library preparation have rarely been explored.

**Results:**

We assessed three different miRNA library preparation protocols, SOLiD, Illumina versions 1 and 1.5, using cloning or SBS sequencing of total RNA samples extracted from skeletal muscles from Hu sheep and Dorper sheep, and then validated 9 miRNAs by qRT-PCR. Our results show that SBS sequencing data highly correlate with Illumina cloning data. The SOLiD data, when compared to Illumina's, indicate more dispersed distribution of length, higher frequency variation for nucleotides near the 3'- and 5'-ends, higher frequency occurrence for reads containing end secondary structure (ESS), and higher frequency for reads that do not map to known miRNAs. qRT-PCR results showed the best correlation with SOLiD cloning data. Fold difference of Hu sheep and Dorper sheep between qRT-PCR result and SBS sequencing data correlated well (r = 0.937), and fold difference of miR-1 and miR-206 among SOLiD cloning data, qRT-PCR and SBS sequencing data was similar.

**Conclusions:**

The sequencing depth can influence the quantitative measurement of miRNA abundance, but the discrepancy caused by it was not statistically significant as high correlation was observed between Illumina cloning and SBS sequencing data. Bias of length distribution, sequence variation, and ESS was observed between data obtained with the different protocols. SOLiD cloning data differ from Illumina cloning data mainly because of distinct methods of adapter ligation. The good correlation between qRT-PCR result and SOLiD data might be due to the similarities of the hybridization-based methods. The fold difference analysis indicated that methods based on hybridization may be superior for quantitative measurement of miRNA abundance. Because of the genome sequence of the sheep is not available, our data may not explain how the entire miRNA bias in the natural miRNAs in sheep or other mammal miRNA expression, unbiased artificially synthesized miRNA will help on evaluating the methodology of miRNA library preparation.

## Background

MicroRNAs(miRNAs) are an abundant group of small RNAs with length ranging from 18 to 25 nucleotides, averaging 22 nucleotides, and performing post-transcriptional regulation of the expression of genes involved in a wide variety of biological processes. The complex biogenesis of mature miRNAs has recently been reviewed [1]. Sequences of more than 10883 miRNAs have been deposited in the miRBase database [2,3], the majority of them having been discovered by traditional cloning approach. Bioinformatics predictions with experimental validation indicate that the total number of miRNAs is significantly higher than previously estimated [4]. It is essential to characterize the whole repertoire of miRNAs and to fully understand their integrated expression patterns. The next-generation sequencing (NGS) techniques enable these efforts with lower cost and have been applied in miRNAs studies in many species of animals, plants and viruses.

Sample preparation is of major importance for NGS and assessing the quality of a library preparation by cloning validation before sequencing is necessary [5]. Different commercial protocols for miRNA library preparation have been developed. Illumina, Inc. published a miRNA sample preparation protocol (V1) for SBS sequencing in 2007, which requires a minimum of 4 days of procedure. In 2009 Illumina, Inc. proposed an alternative protocol (V1.5) which only requires one day of sample preparation. Applied Biosystems, Inc. developed a miRNA library preparation protocol for the SOLiD (Sequencing by Oligonucleotide Ligation and Detection) system, also requiring one day procedure, but its adapter ligation principle is based on hybridization. These protocols can be applied in all current sequencing techniques though the downstream procedures can be variable.

The cloning frequency of an individual miRNA should generally reflect its relative abundance in a sample, and the novel NGS methods offering a much richer source of sequence information should provide more accurate quantitative expression measurements [6]. However, in reality biases caused by sample preparation cannot be avoided, sometimes leading to inaccurate conclusions. A systematic bias in the cloning protocol has previously been detected: miRNA clone counts did not correlate well with their concentrations in the pool [7]. Biased cloning efficiencies were also observed for two different miRNAs from the same cluster, leading to discrepancies between cloning frequency and small RNA blot results [8].

Different protocols of library preparation may influence the cloning frequency significantly. The adapter ligation efficiency can be affected by the 5'- and 3'-end nucleotides or the secondary structure of miRNAs, and the number of polymerase chain reaction cycles or gel isolation procedures may also influence the results. In this article we compared sequencing data of libraries constructed by the above-mentioned three different protocols, and validated some results by qRT-PCR using stem-loop primers [9]. Bias of length, sequence variation, and ESS were observed for all three protocols. Based on our data, we suggest that methods such as SOLiD and qRT-PCR, based on hybridization, may provide better quantitative measurement of miRNA abundance.

## Results and discussion

### Statistics for cloning and Illumina SBS sequencing libraries

**Figure 1:**
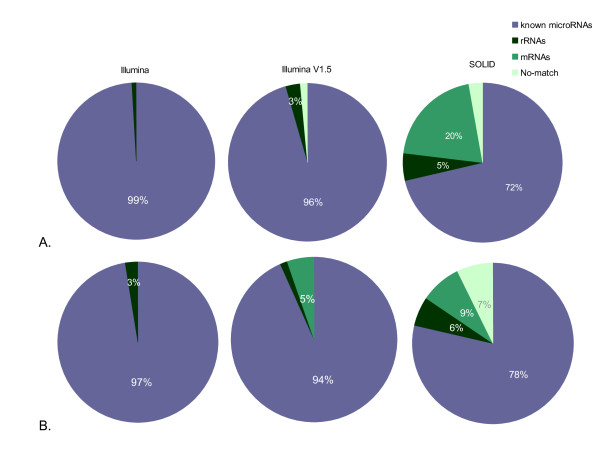
**Annotation for cloning data**. The count of reads mapping to known microRNAs (slate), rRNAs (dark green), to mRNAs (green) or not mapping to any known sequences (No-match, light green) were assessed for cloning data of Dorper sheep (A) and Hu sheep (B), respectively. The data were obtained by sequencing of libraries constructed by protocols of Illumina V1, Illumina V1.5, and SOLiD, respectively.

We assessed the quality of libraries by cloning and a Sanger sequencing approach. 211, 228 and 233 high quality reads were obtained for Hu sheep libraries constructed by SOLiD, Illumina V1 and Illumina V1.5 protocols, respectively. 221, 225 and 208 high quality reads were obtained for Dorper sheep using SOLiD, Illumina V1 and Illumina V1.5 protocols, respectively. The ratio of reads mapping to known microRNAs, rRNAs, and mRNAs, and the reads which have not been mapped to any known sequences, were compared between the different protocols (Figure 1).

The libraries constructed from Dorper sheep and Hu sheep, using the Illumina V1 protocol, were used for Illumina SBS sequencing. About 6 million raw reads were obtained for each library. Eighty-four percent of the Hu sheep reads mapped to known *Ovis aries *sequences and 111,078 were unique reads for Hu sheep. Concerning Dorper sheep, 82% of the reads could map to known *Ovis aries *sequences, and 147,044 unique reads were observed. About 5.8 and 5.6 million reads were obtained after adapter removing for Dorper sheep and Hu sheep respectively. Of these reads 82% and 84%, respectively, have previously been annotated as either known RNAs (rRNA, tRNA, snRNA etc, incl. miRNA), repeat regions, or are contained within the boundaries of protein coding genes for Dorper sheep and Hu sheep. The number of reads that were annotated as known microRNAs was 4,812,498 and 4,904,192 for Dorper sheep and Hu sheep respectively.

### Length distribution for libraries

**Figure 2:**
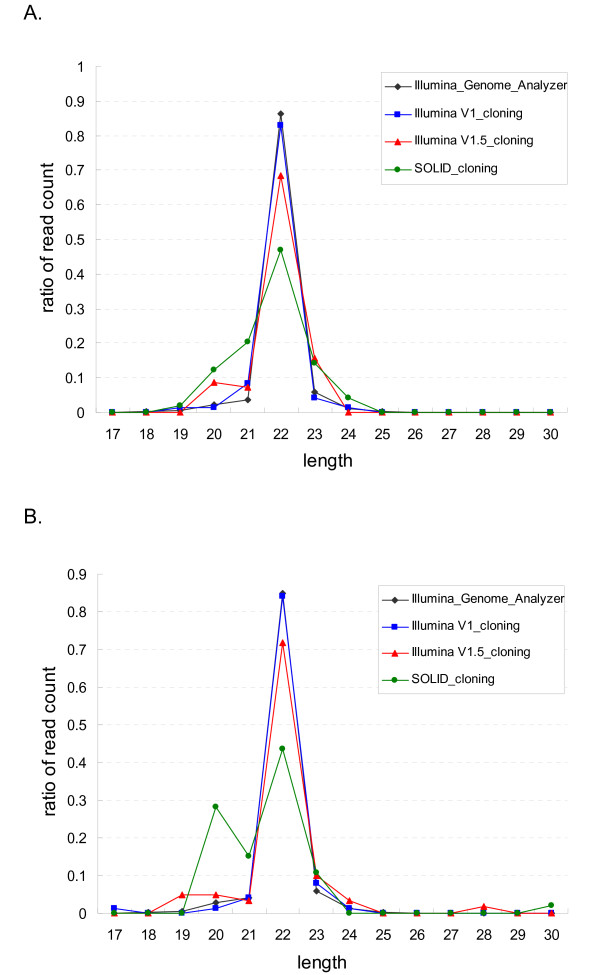
**Length distribution of Hu sheep and Dorper sheep sequencing data**. Length distribution of sequencing data of libraries by Illumina Genome Analyzer (black), Illumina V1 cloning (blue), Illumina V1.5 cloning (red), SOLiD cloning (green) for Hu sheep (A) and Dorper sheep (B) are shown. X-axis corresponds to the length, Y-axis to the ratio of count of reads for specific length.

The length distribution of all cloning and SBS sequencing data was assessed (Figure 2). They follow a Gaussian-like distribution with the mean set at 22 nt. Kurtosis of each distribution curve varies according to the protocols. The SOLiD protocol covers a wider range of length, as the gradient of the distribution curve is flatter. The Illumina protocols have intensive enriching effects for 22 nt miRNAs, the V1 protocol showing the highest effect. A more strict length distribution may be obtained by introducing additional gel purification steps in the protocols. Length distribution of Illumina Genome Analyzer (GA) data (SBS sequencing) coincided with Illumina V1 cloning data as the same library preparation protocol was used.

### Different protocols generate different bias of sequence variation and end secondary structure (ESS)

The sequence variations of miR-1 and miR-206 in our data were assessed by WebLogo tool (Additional file 1, 2, 3, 4, 5, and 6). The sequences obtained by SOLiD cloning display a higher diversity than the ones from Illumina. We observed obvious higher-frequency variation of nucleotides near 3'-end sites. The adenine and thymine at the 3'-end of miR-1 were truncated in the majority of SOLiD cloning sequences. Variations of 5'-end nucleotides were also found in SOLiD cloning data, but were rare in all the data from Illumina protocols. Comparing the sequences obtained by the two Illumina protocol versions, we observed a generally high conservatism, though nucleotides near the 3'-end showed slightly more diversity using Illumina V1.5 protocol.

The 16, 17 sites of miR-1 have lower conservatism and more diversity for SOLiD cloning sequences (Additional file 1 and 4), this phenomenon can also be observed at the 17, 18 sites of miR-206 in SOLiD data, but not in Illumina's (Additional file 2 and 5). The sites listed above all locate near the 3'-end, however, the sites near the 5'-end indicated high conservatism for both SOLiD and Illumina data. Diversity of these sites near the 3'-end may be caused by the hybridization-based adapter ligation in the SOLiD protocol.

**Figure 3:**
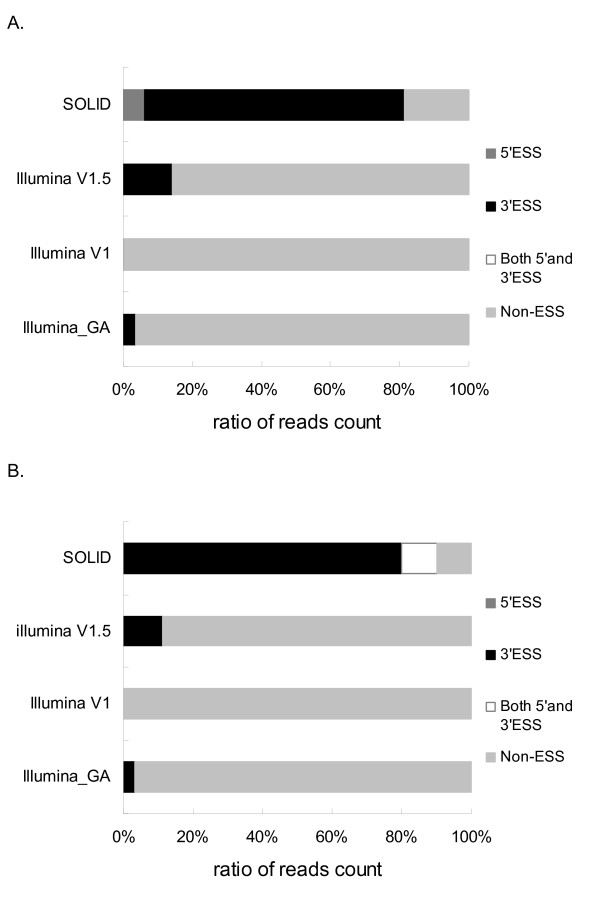
**End Secondary Structure (ESS) of miR-1 sequences in Dorper sheep and Hu sheep data**. Reads mapping to miR-1 sequence from Illumina GA data, Illumina V1 cloning, Illumina V1.5 cloning, and SOLiD cloning data of Dorper sheep (A) and Hu sheep (B) were assessed by RNA mfold software. The ratio of reads containing secondary structure at 5'- or/and 3'-end, or containing secondary structure within the sequence (non-ESS) were shown.

Bias of ESS for miR-1 sequences were observed between SOLiD and Illumina protocols (Figure 3). The presence of ESS at 5'- or 3'-ends was highly uneven. The frequency of 5'-ESS was much lower than 3'-ESS for miR-1 sequences, which may be due to lower-frequency variation in 5'-end sequences. In the SOLiD cloning data 75% and 80% of the miR-1 sequences contained 3'-ESS for Dorper sheep and Hu sheep, respectively. Conversely, the 3'-ESS sequences accounted for less than 15% of the Illumina data. 5'-ESS sequences were rather rare in the Illumina GA data (0.16%), and were absent in the Illumina cloning data with the two version protocols due to limited counts of reads, but 5'-ESS accounted for 10% and 6.3% for SOLiD cloning data of Hu sheep and Dorper sheep, respectively. The SOLiD data contained significantly more ESS than Illumina's, which may be caused by an enrichment effect on ESS using the SOLiD protocol, or an enrichment effect on non-ESS using the Illumina protocol.

Bias of ESS also existed between the two versions of Illumina protocols (Figure 3). Data acquired with the V1.5 protocol contained more than 10% 3'-ESS for miR-1 sequences, while the data of the V1 protocol did not contain any ESS for miR-1 sequences.

### Relative abundance of miRNAs varied for different protocols

We sorted the reads mapping to known microRNAs into different families, assessing the read counts of each miRNA family by correlative analysis. For Dorper sheep, we obtained sequences from 199 miRNA families for Illumina SBS sequencing while only 26 families were observed when combining the results from the three libraries for cloning and Sanger sequencing (Additional file 7 and 8), indicating a high depth of SBS sequencing. The 26 families mentioned above were analyzed in SBS and Sanger sequencing data, and the same procedure was used for Hu sheep. The sequencing data all correlate significantly, but the correlation coefficients varied for different protocols. The correlation coefficients between SOLiD and Illumina data were the lowest, regardless of SBS or Sanger sequencing, and the Illumina GA data correlated best with Illumina cloning data because the same protocol was used (0.944 and 0.949 for Dorper sheep and Hu sheep, respectively) (Table 1).

**Table 1:** Correlation coefficients between qRT-PCR and sequencing data for Dorper sheep and Hu sheep

		SOLiD	lllumina V1	lllumina V1.5	lllumina GA
	qRT-PCR	0.9399	0.9118	0.9036	0.9178
Dorper sheep	Lllumina V1	0.7785	0.9441	0.9427	
	Lllumina V1	0.7797	0.9478		
Hu sheep	Lllumina V1	0.8400			
	qRT-PCR	0.9543	0.9180	0.9246	0.9474
	Lllumina GA	0.8818	0.9491	0.9172	
	Lllumina V1.5	0.8639	0.9244		
	Lllumina V1	0.9105			

Correlation coefficients between qRT-PCR and sequencing data were calculated by the data listed in Additional File 10. Correlation coefficients between different sequencing data were calculated by the data listed in Additional File 7.

A relatively higher frequency of sequences that do not map to known miRNAs was observed for SOLiD cloning data (about 20%), including the sequences mapping to mRNAs, repeats, or rRNA genes, and sequences that do not map to any known sequences (Figure 1).

### qRT-PCR results correlate the best with SOLiD cloning data

**Figure 4:**
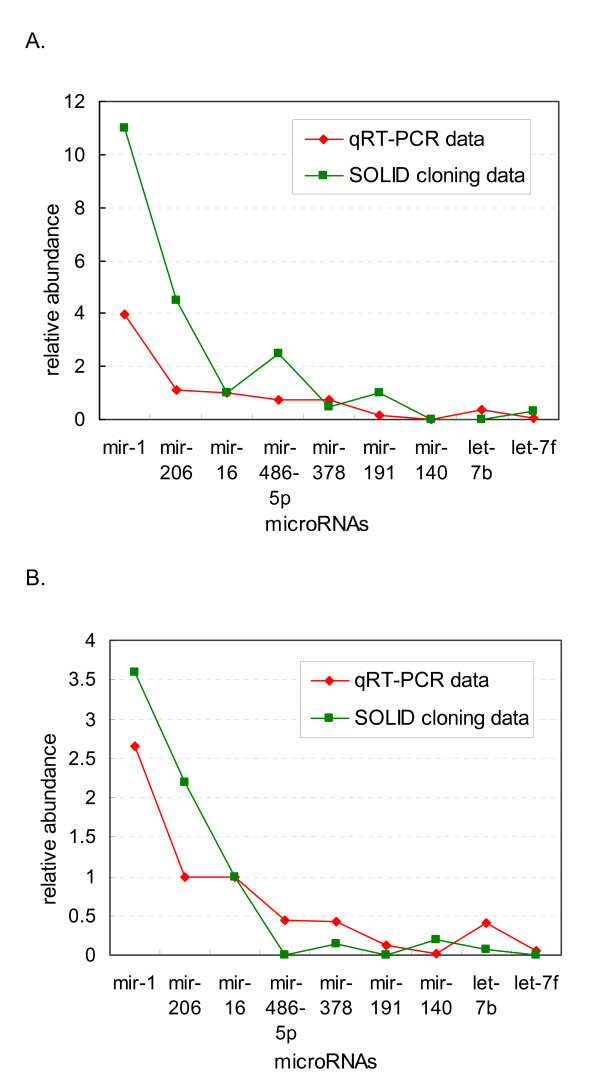
**qRT-PCR and SOLiD cloning data for Hu sheep and Dorper sheep**. The relative abundance of 9 miRNAs calculated by qRT-PCR (red) and SOLiD cloning sequencing (green) for Hu sheep (A) and Dorper sheep (B) are shown. Relative abundance of the miRNAs was calculated by the 2^-ΔΔCT ^method using miR-16 as reference to normalize the results. Relative abundance of the miRNAs for the SOLiD cloning data was calculated by the frequency of reads count of each miRNA.

The relative abundance of 9 miRNAs calculated by qRT-PCR was compared with the read counts of the same families in sequencing data. The muscle-specific miRNAs, miR-1 and miR-206, were the two most abundant miRNAs as analyzed by qRT-PCR, and confirmed by sequencing data, as well as by a previous report [10]. Sequencing and qRT-PCR data all significantly correlated, displaying correlation coefficients above 0.9 (Table 1). Correlation coefficients between SOLiD cloning data and qRT-PCR were the highest (0.954 and 0.94 for Hu sheep and Dorper sheep, respectively) (Table 1 and Figure 4). The correlation between Illumina GA data and qRT-PCR were also excellent (0.947 and 0.918, respectively). The high correlation between data from qRT-PCR and SOLiD protocol may be explained by the similar principle of hybridization between the miRNA templates and the stem-loop primers or the adapter mix.

### Fold difference analysis between Hu sheep and Dorper sheep using qRT-PCR and sequencing

**Figure 5:**
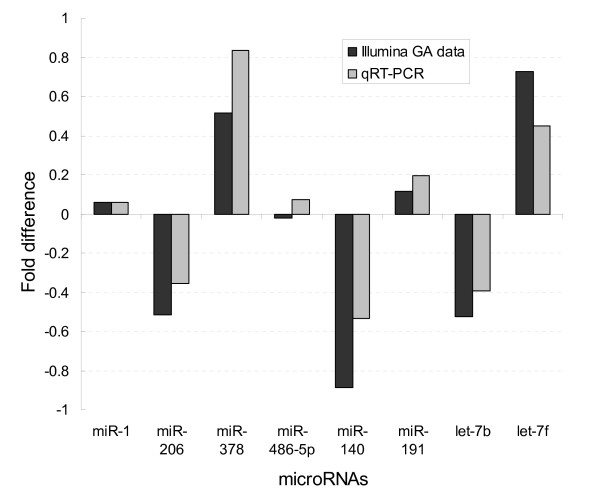
**Fold difference of qRT-PCR and Illumina GA data between Hu sheep and Dorper sheep**. Fold difference between Hu sheep and Dorper sheep of qRT-PCR (grey) and Illumina GA data (black) for 9 miRNAs are shown. Fold differences for qRT-PCR data were calculated by ΔΔCT between Hu sheep and Dorper sheep, where ΔΔCT = ΔCT_miRNA_-ΔCT_miR-16_. Fold differences for Illumina GA data were calculated by the equation: log2 (ratio of read count of Hu sheep/ratio of read count of Dorper sheep).

**Figure 6:**
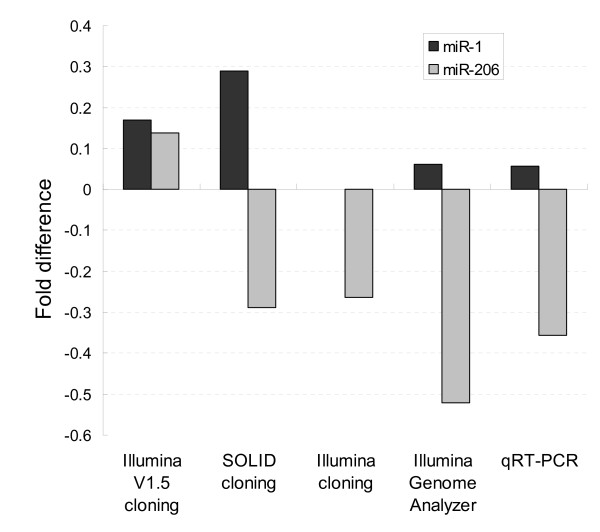
**Fold difference of miR-1 and miR-206 between Hu sheep and Dorper sheep**. Fold differences of muscle-specific miRNAs, miR-1 and miR-206, between Hu sheep and Dorper sheep were assessed amongst cloning and SBS sequencing data and qRT-PCR. Fold differences for qRT-PCR data were calculated by ΔΔCT between Hu sheep and Dorper sheep, where ΔΔCT = ΔCT_miRNA_-ΔCT_miR-16_. Fold differences for sequencing data were calculated by the equation: log2 (ratio of reads count of Hu sheep/ratio of reads count of Dorper sheep).

Except for miR-486-5p, the miRNAs assessed showed a similar orientation of fold change using qRT-PCR or Illumina GA (Figure 5). The correlation coefficient between the fold difference of the 8 miRNAs calculated by qRT-PCR and Illumina GA was 0.937. Fold difference of miR-1 and miR-206 for cloning data using different protocols were compared with SBS sequencing data and qRT-PCR (Figure 6). Fold difference of miR-206 was inverted for Illumina V1.5 cloning data, and fold difference of miR-1 could not be detected by cloning using Illumina V1 protocol. For SOLiD cloning data, the fold difference of miR-1 and miR-206 was similar with Illumina GA data and qRT-PCR, indicating relatively more accurate quantitative measurement for miRNA abundance of the SOLiD protocol.

## Conclusions

In the present study, we assessed three different protocols of miRNA library construction using cloning or SBS sequencing, and validated our results by qRT-PCR. SBS sequencing provided a high-throughput and deep measurement for miRNA expression, while the sequencing depth of cloning was much lower, though a concatemerization cloning strategy was developed [11]. SBS sequencing data correlated better with qRT-PCR results than did Illumina cloning data, indicating that sequencing depth would influence the quantitative measurement of miRNA abundance, but the discrepancy caused by it was not significant, as seen from the high correlation between SBS and Illumina cloning data.

**Figure 7:**
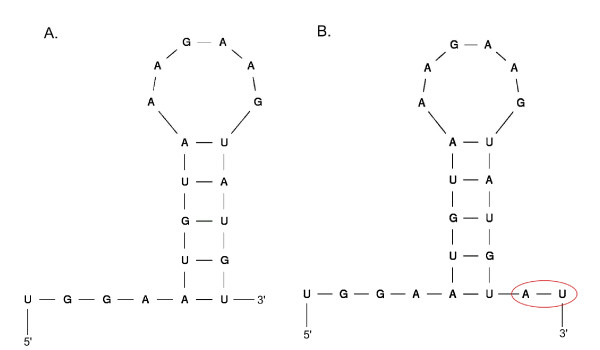
**Structure of original and truncated sequences of miR-1**. Structures of two miR-1 sequences that appeared in SOLiD cloning data are shown. The left part of the figure represents the truncated sequence (A), and the right part represents the original sequence (B). 3'- ESS appears on the truncated sequence, while the original sequence contains secondary structures which are not located on the ends.

Bias of sequence variation, end secondary structure (ESS), and length distribution was observed for the different protocols of library construction. The SOLiD data differ from the Illumina data, due to distinct principles of adapter ligation in protocol. The adapter ligation principle based on 6 random nucleotides (N6) hybridization seemed to provide a more dispersed distribution of length, higher frequency variation for nucleotides near the 3'- or 5'-ends, higher frequency of reads containing ESS, and higher frequency of reads which do not map to known miRNAs in sequencing data. The two nucleotides at the 3'-end of the majority of the reads of miR-1 in SOLiD cloning data were truncated, which may be due to RNA editing. We also found that the truncated sequence of miR-1 can form 3'-ESS while the original sequence can not (Figure 7). The end secondary structures can hide the 5'- or 3'- end nucleotide, so that the direct adapter ligation may fail, but the pre-hybridization step preceding ligation may eliminate the ESS, enabling subsequent successful ligation. That can explain the high-frequency of ESS occurring in the SOLiD cloning data. We also observed more than 10% 3'-ESS for miR-1 sequences in Illumina V1.5 cloning data, but no ESS for the Illumina V1 protocol, indicating that the T4 RNA ligase 2 (Rnl2) used in V1.5 protocol may enable more adapters to ligate to miRNAs with a double-stranded structure at the 3'-end [12].

We finally assessed the relative abundance of 9 miRNAs by qRT-PCR. The principle of reverse transcription (RT)-PCR with stem-loop primer is based on hybridization as for the SOLiD protocol, which could explain the high correlation between qRT-PCR and SOLiD cloning data. The fold difference data between Hu sheep and Dorper sheep using qRT-PCR and SBS sequencing correlated significantly, and the fold difference data for miR-1 and miR-206 using SOLiD cloning were similar to data obtained with SBS sequencing and qRT-PCR, indicating that the methods using a hybridization principle may be more suitable for quantitative measurement of miRNA abundance. Moreover, qRT-PCR has been used prevalently for validation of microarray results [13,14] and its accuracy has been recognized.

## Methods

### Total RNA preparation and DNase I treatment, Isolation of small RNAs

Total RNA from skeletal muscle tissues of Hu sheep and Dorper sheep were extracted using Trizol (Invitrogen, Carlsbad, CA) according to the manufacturer's protocol. About 10 ug total RNA was treated with DNase I (NEB) and then purified by ethanol precipitation.

### MiRNA libraries construction

**Figure 8:**
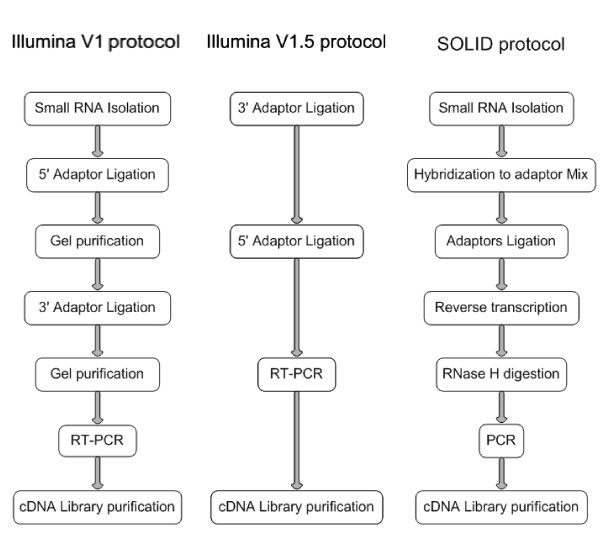
**Flow-sheet of protocols**. Flow-sheet of Illumina V1, Illumina V1.5 and SOLiD protocols for miRNA library construction are shown.

In Illumina V1 protocol, isolated 18-30 nt small RNAs were first ligated with 5' adapter using T4 RNA ligase. Ligation products were gel-purified, then ligated with 3' adapter using T4 RNA ligase and purified. Small RNAs with adapters on both ends were used as templates for reverse transcription PCR to create cDNA constructs. The amplified cDNA constructs were subsequently purified and used for loading on an Illumina Cluster Station (Figure 8).

The Illumina V1.5 protocol has been previously described [15]. Pre-adenylated 3' adapter deoxyoligonucleotides were used, and their 3' ends blocked. A truncated form of T4 RNA ligase 2, Rnl2, was used for 3' adapter ligation without ATP. Then 5' adapter, ATP, and T4 RNA ligase were added to the ligation mix without purification. Then reverse transcription PCR was performed to create and amplifiy cDNA constructs, which were then gel-purified.

The SOLiD protocol allows simultaneous 5' and 3' adapter ligation to the ends of small RNAs, a method based on hybridization of N6 at the end of the adapters. After ligation of adapters, reverse transcription was performed followed by RNase H digestion and cDNA library amplification. The library was finally size-selected and purified.

### Cloning and SBS sequencing

Cloning of miRNAs was performed as described previously [16]. The resulting cDNA libraries following the three protocols for Hu sheep and Dorper sheep were cloned and transformed into competent cells. Plasmids were isolated from individual colonies and sequenced. The sequences were subsequently processed to remove vector sequences and used for BLASTN analysis against the miRBase database [2,3].

SBS sequencing using Illumina Genome Analyzer was performed for cDNA libraries of Hu sheep and Dorper sheep constructed by Illumina V1 protocol. 10 pM of each sample was used for cluster generation. After hybridization of sequencing primer, 35 cycles of base incorporation were carried out on the 1 G analyzer. Image analysis and basecalling were performed using Illumina Pipeline. The sequence tags obtained after purity filtering were sorted and annotated. The reads mapping to known miRNAs were annotated using the miRBase database [17].

### Prediction of End Secondary Structure (ESS) for miR-1 and miR-206

We predicted the secondary structure for the reads of miR-1 in sequencing data by an RNA mfold web server using default parameters [18,19]. Whenever a stem-loop structure was able to be formed in the 5'- or 3'-end of an miRNA sequence so that a terminal double-stranded structure would appear, we considered this sequence as containing ESS. We counted the reads with and without either 5'- ESS or 3'- ESS, or both.

### Assessment of sequence variation of miR-1 and miR-206 by WebLogo

We assessed the variation of miR-1 and miR-206 sequences in our data by a WebLogo tool [20]. In order to reflect the variation of 3'-end sequences, we added "N" to the vacancy sites at the 3'-end to bring all the sequences to the same length. miR-1 and miR-206 sequences of cloning and Illumina GA data were all assessed.

### Real-time quantitative RT-PCR (qRT-PCR)

We selected the following nine miRNAs including miR-1, miR-206, miR-378, miR-486-5p, miR-140, miR-191, miR-16, let-7b, and let-7f. miR-16 was used as reference. The sequences of the primers are listed in Additional file 9. Stem-loop primers were preheated at 95°C for 3 min, then gradually cooled down to room temperature. 10 ng purified total RNA was used as template for a total of 10 ul reaction. 10 nM of each miRNA specific reverse transcription primer together with 10 U RNase Out, 5U Superscript II, 5 mM DTT and 20 mM dNTP were used for each RT reaction. Samples were incubated at 16°C for 30 min, then at 42°C for 30 min, and finally at 75°C for 15 min to inactivate the Superscript II enzyme.

Four microlitres of RT product were used as template for a 20 ul reaction of real-time PCR. All reactions were assayed in triplicates. Real-time PCR was performed using a TaKaRa SYBR Premix Ex Taq kit according to the manufacturer's protocol on an Applied Biosystems StepOnePlus Real-time PCR System. The reaction conditions were modified as follows: 95°C for 30 sec, followed by 40 cycles of 5 sec at 95°C, and 63°C for 31 sec. miR-16 was used to normalize the results. The relative abundance of miRNAs and fold difference between Hu sheep and Dorper sheep were calculated using the 2^-ΔΔCT ^method.

### Fold difference analysis using qRT-PCR and sequencing data

The ΔΔCT of 8 miRNAs between DNase-treated total RNA samples of Hu sheep and Dorper sheep were calculated with miR-16 as endogenous reference to normalize the qRT-PCR result. The log ratio of read counts of the same 8 miRNAs between Illumina GA data of Hu sheep and Dorper sheep were calculated. For limited sequencing depth, some low-abundance miRNAs cannot be detected by cloning. We selected the two muscle-specific miRNAs, miR-1 and miR-206, which were abundant in all sequencing data, and calculated the read counts log ratio of these two miRNAs between the data of Hu sheep and Dorper sheep. The fold difference of Hu sheep and Dorper sheep between qRT-PCR and sequencing data were compared (Additional file 10).

## Competing interests

The authors declare that they have no competing interests.

## Authors' contributions

GT participated in the design of the study. XYY participated in the design of the study, drafted the manuscript, and carried out the qRT-PCR studies. HL and XHX carried out miRNA libraries construction for cloning and SBS sequencing. ZXQ participated in the design of the study. LARS helped revising the manuscript. All authors read and approved the final manuscript.

## Supplementary Material

Additional file 1 **WebLogo for miR-1 reads of Hu sheep**. The miR-1 reads from Illumina GA data, Illumina V1 cloning, Illumina V1.5 cloning, and SOLiD cloning data of Hu sheep were assessed by WebLogo tool. Click here for file

Additional file 2 **WebLogo for miR-206 reads of Hu sheep**. The miR-206 reads from Illumina GA data, Illumina V1 cloning, Illumina V1.5 cloning, and SOLiD cloning data of Hu sheep were assessed by WebLogo tool. Click here for file

Additional file 3 **WebLogo for miR-1 and miR-206 reads of Hu sheep**. The miR-1 and miR-206 reads from Illumina GA data, Illumina V1 cloning, Illumina V1.5 cloning, and SOLiD cloning data of Hu sheep were assessed by WebLogo tool. Click here for file

Additional file 4 **WebLogo for miR-1 reads of Dorper sheep**. The miR-1 reads from Illumina GA data, Illumina V1 cloning, Illumina V1.5 cloning, and SOLiD cloning data of Dorper sheep were assessed by WebLogo tool. Click here for file

Additional file 5 **WebLogo for miR-206 reads of Dorper sheep**. The miR-206 reads from Illumina GA data, Illumina V1 cloning, Illumina V1.5 cloning, and SOLiD cloning data of Dorper sheep were assessed by WebLogo tool. Click here for file

Additional file 6 **WebLogo for miR-1 and miR-206 reads of Dorper sheep**. The miR-1 and miR-206 reads from Illumina GA data, Illumina V1 cloning, Illumina V1.5 cloning, and SOLiD cloning data of Dorper sheep were assessed by WebLogo tool. Click here for file

Additional file 7 **Read counts of miRNA families for cloning sequencing and Illumina GA data**. Read counts of miRNA families for cloning data of Hu sheep and Dorper sheep are shown together with Illumina GA data of the corresponding miRNA families. Click here for file

Additional file 8 **Read counts of miRNA families for Illumina GA data**. Read counts of all miRNA families for Illumina GA data (SBS sequencing) of Hu sheep and Dorper sheep are shown. The miRNA ID and accession No of each miRNA family have been listed. Click here for file

Additional file 9 **Primer sequences for qRT-PCR**. Sequences of RT stem-loop primers and forward/reverse primers of 9 miRNAs for qRT-PCR. Click here for file

Additional file 10 **The qRT-PCR result and read counts for 9 miRNAs**. Relative abundance for 9 miRNAs calculated by qRT-PCR are shown, with the read counts of sequencing data for these 9 miRNAs. Click here for file

## References

[B1] Winter Julia Jung Stephanie Keller Sarina Gregory Richard I Diederichs Sven Many roads to maturity: microRNA biogenesis pathways and their regulation Nature Cell Biol 2009 11 228 234 10.1038/ncb0309-228 19255566

[B2] Griffiths-Jones S Grocock RJ van Dongen S Bateman A Enright AJ MiRBase: microRNA sequences, targets and gene nomenclature Nucleic Acid Res 2006 34 D140 D144 10.1093/nar/gkj112 16381832 PMC1347474

[B3] The miRBase Database http://www.mirbase.org/

[B4] Xie X Lu J Kulbokas EJ Golub TR Mootha V Lindblad-Toh K Lander ES Kellis M Systematic discovery of regulatory motifs in human promoters and 3' UTRs by comparison of several mammals Nature 2005 434 338 345 10.1038/nature03441 15735639 PMC2923337

[B5] Lu Cheng Meyers Blake C Green Pamela J Construction of small RNA cDNA libraries for deep sequencing Methods 2007 43 110 117 10.1016/j.ymeth.2007.05.002 17889797

[B6] Bar M Wyman SK Fritz BR Tewari M MicroRNA Discovery and Profiling in Human Embryonic Stem Cells by Deep Sequencing of Small RNA Libraries Stem Cells 2008 26 2496 2505 10.1634/stemcells.2008-0356 18583537 PMC2847579

[B7] Landgraf Pablo Rusu Mirabela Sheridan Robert A mammalian microRNA expression atlas based on small RNA library sequencing Cell 2007 129 1401 1414 10.1016/j.cell.2007.04.040 17604727 PMC2681231

[B8] Reddy Alavala Matta Zheng Yun Jagadeeswaran Guru Macmil Simone L Graham Wiley B Roe Bruce A Desilva Udaya Zhang Weixiong Sunkar Ramanjulu Cloning, characterization and expression analysis of porcine microRNAs BMC Genomics 2009 10 1471 2164 10.1186/1471-2164-10-65 PMC2644714 19196471

[B9] Chen Caifu Ridzon Dana A Broomer Adam J Real-time quantification of microRNAs by stem-loop RT-PCR Nucleic Acids Res 2005 33 e179 10.1093/nar/gni178 16314309 PMC1292995

[B10] Callis Thomas E Deng ZhongLiang Chen Jian-Fu Wang Da-Zhi Muscling through the microRNA world Exp Biol Med(Maywood) 2008 233 131 138 10.3181/0709-MR-237 18222968

[B11] Pfeffer Sebastien Lagos-Quintana Mariana Tuschl Thomas Cloning of small RNA molecules Current Protocols in Molecular Biology 2005 26.4.1 26.4.18 10.1002/0471142727.mb2604s72 18265364

[B12] Nichols NM Tabor S McReynolds LA RNA ligase Curr Protoc Mol Biol 2008 Chapter 3 Unit3.15 10.1002/0471142727.mb0315s84 18972386

[B13] Bruchova Hana Merkerova Michaela Prchal Josef T Aberrant expression of microRNA in polycythemia vera Haematologica 2008 93 1009 1016 10.3324/haematol.12706 18508790

[B14] Gibcus Johan H Tan Lu Ping Harms Geert Schakel Rikst Nynke de Jong Debora Blokzijl Tjasso Moller Peter Poppema Sibrand Kroesen Bart-Jan van den Berg Anke Hodgkin lymphoma cell lines are characterized by a specific miRNA expression profile Neoplasia 2009 11 167 176 19177201 10.1593/neo.08980 PMC2631141

[B15] Hafner Markus Landgraf Pablo Ludwig Janos Identification of microRNAs and other small regulatory RNAs using cDNA library sequencing Method 2008 44 3 12 10.1016/j.ymeth.2007.09.009 PMC2847350 18158127

[B16] Sunkar Ramanjulu Girke Thomas Jain Pradeep Kumar Zhu Jian-Kang Cloning and characterization of microRNAs from rice The Plant Cell 2005 17 1397 1411 10.1105/tpc.105.031682 15805478 PMC1091763

[B17] Ambros V Bartel B Bartel DP Burge CB Carrington JC Chen X Dreyfuss G Eddy SR Griffiths-Jones S Marshall M Matzke M Ruvkun G Tuschl T A uniform system for microRNA annotation RNA 2003 9 277 279 10.1261/rna.2183803 12592000 PMC1370393

[B18] Zuker M Mfold web server for nucleic acid folding and hybridization prediction Nucleic Acid Res 2003 31 3406 15 10.1093/nar/gkg595 12824337 PMC169194

[B19] Mathews DH Sabina J Zuker M Turner DH Expanded sequence dependence of thermodynamic parameters improves prediction of RNA secondary structure J Mol Biol 1999 288 911 940 10.1006/jmbi.1999.2700 10329189

[B20] Crooks Gavin E Hon Gary Chandonia John-Marc Brenner Steven E WebLogo: A sequence logo generator Genome Research 2004 14 1188 1190 10.1101/gr.849004 15173120 PMC419797

