# Supplementary figures and images for: Sequencing bias: comparison of different protocols of MicroRNA library construction
Source: BMC Biotechnol. 2010 Sep 6;10:64. doi: 10.1186/1472-6750-10-64 (PMC2946280; doi:10.1186/1472-6750-10-64)

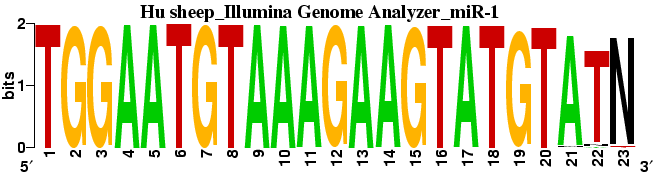


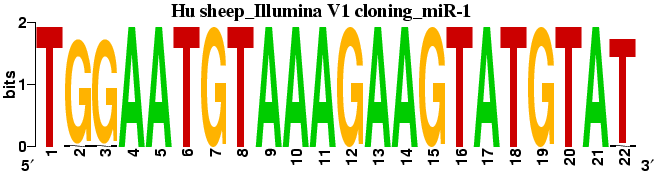


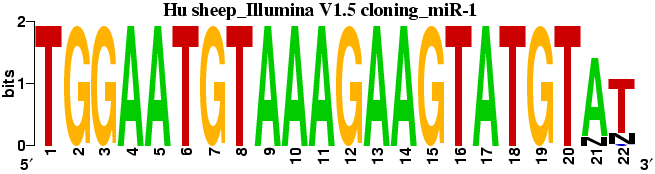


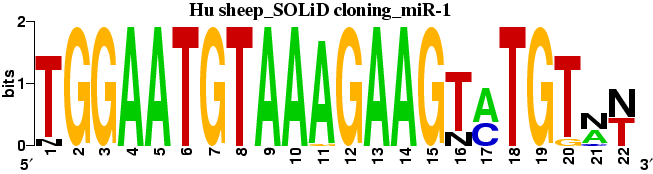

Supplement: Additional file 1 — WebLogo for miR-1 reads of Hu sheep. The miR-1 reads from Illumina GA data, Illumina V1 cloning, Illumina V1.5 cloning, and SOLiD cloning data of Hu sheep were assessed by WebLogo tool. [file 1472-6750-10-64-S1.DOC]

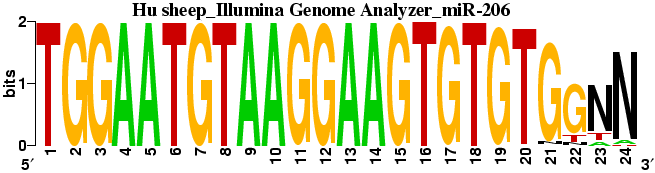


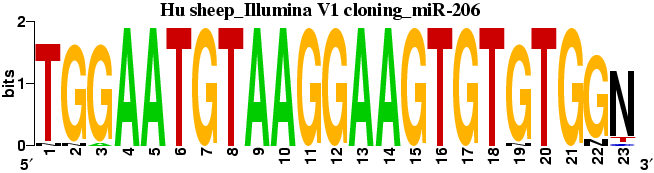


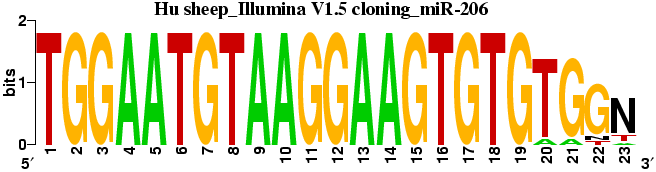


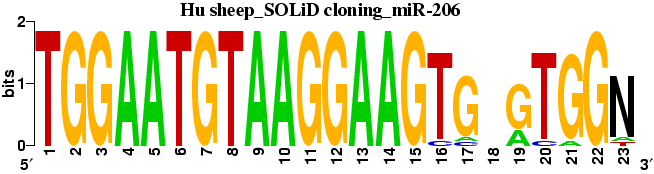

Supplement: Additional file 2 — WebLogo for miR-206 reads of Hu sheep. The miR-206 reads from Illumina GA data, Illumina V1 cloning, Illumina V1.5 cloning, and SOLiD cloning data of Hu sheep were assessed by WebLogo tool. [file 1472-6750-10-64-S2.DOC]

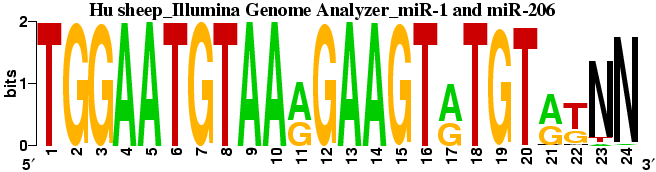


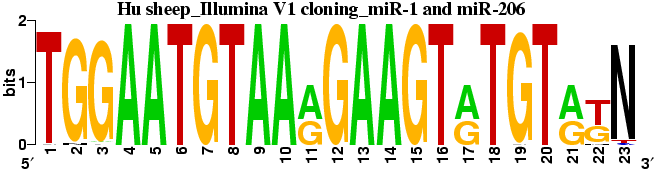


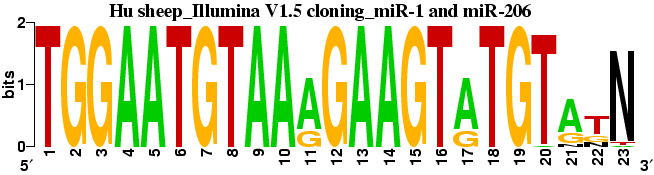


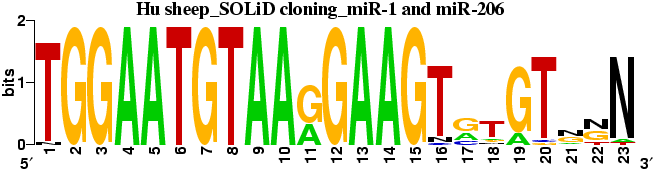

Supplement: Additional file 3 — WebLogo for miR-1 and miR-206 reads of Hu sheep. The miR-1 and miR-206 reads from Illumina GA data, Illumina V1 cloning, Illumina V1.5 cloning, and SOLiD cloning data of Hu sheep were assessed by WebLogo tool. [file 1472-6750-10-64-S3.DOC]

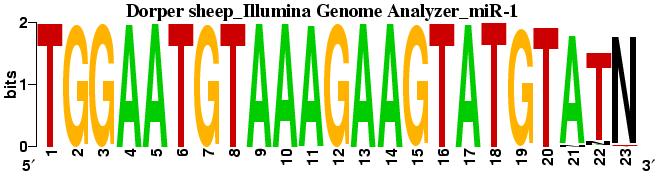


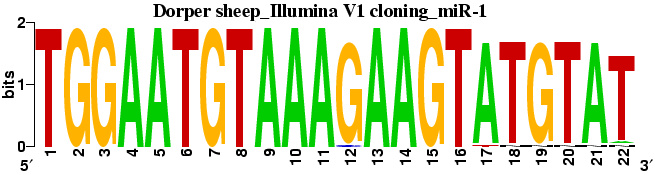


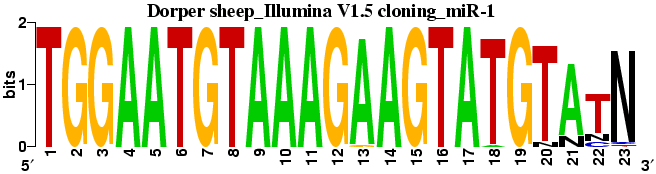


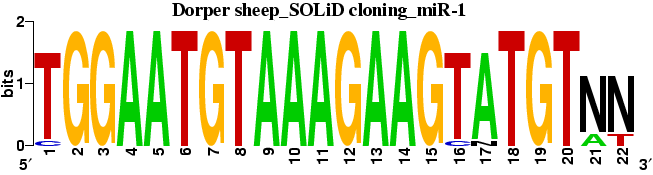

Supplement: Additional file 4 — WebLogo for miR-1 reads of Dorper sheep. The miR-1 reads from Illumina GA data, Illumina V1 cloning, Illumina V1.5 cloning, and SOLiD cloning data of Dorper sheep were assessed by WebLogo tool. [file 1472-6750-10-64-S4.DOC]

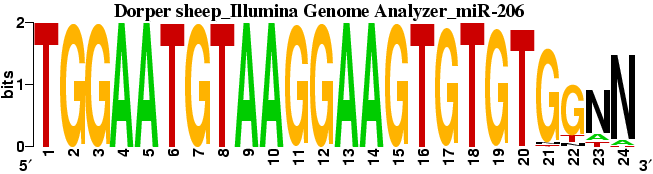


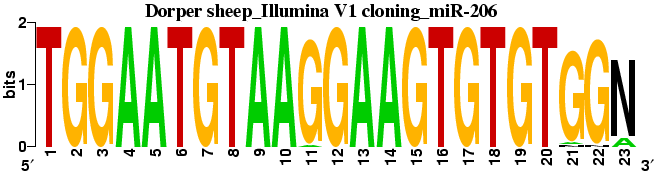


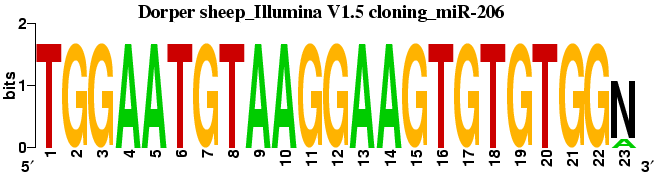


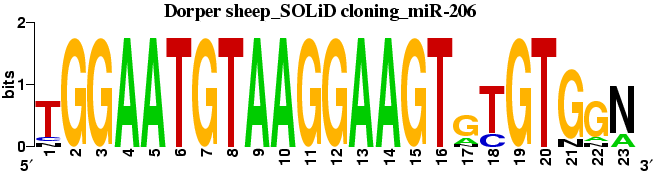

Supplement: Additional file 5 — WebLogo for miR-206 reads of Dorper sheep. The miR-206 reads from Illumina GA data, Illumina V1 cloning, Illumina V1.5 cloning, and SOLiD cloning data of Dorper sheep were assessed by WebLogo tool. [file 1472-6750-10-64-S5.DOC]

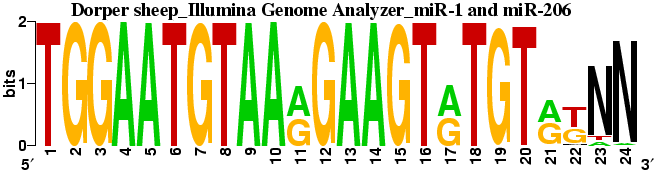

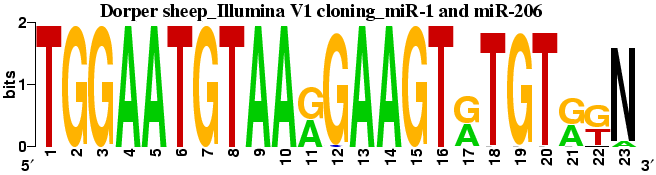

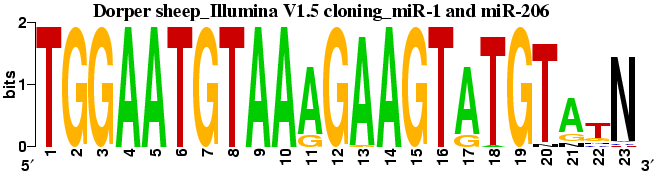


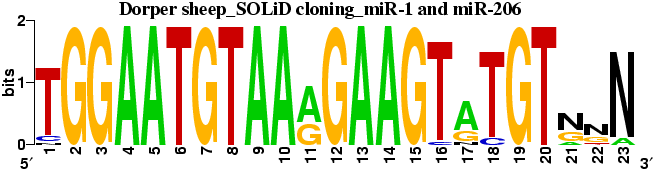

Supplement: Additional file 6 — WebLogo for miR-1 and miR-206 reads of Dorper sheep. The miR-1 and miR-206 reads from Illumina GA data, Illumina V1 cloning, Illumina V1.5 cloning, and SOLiD cloning data of Dorper sheep were assessed by WebLogo tool. [file 1472-6750-10-64-S6.DOC]
